# Adult Presentation of Subaortic Stenosis with Subaortic Membrane Treated with Surgical Removal

**DOI:** 10.3390/jcdd9020036

**Published:** 2022-01-21

**Authors:** Se Hun Kang, In Jai Kim, Won-Jang Kim

**Affiliations:** CHA Bundang Medical Center, Department of Cardiology, CHA University, Seongnam 13496, Korea; kirara00@gmail.com (S.H.K.); injaikim@cha.ac.kr (I.J.K.)

**Keywords:** aortic valve stenosis, aortic stenosis, subvalvular

## Abstract

Subaortic stenosis (SAS) is a rare heart disease in adults with an unclear etiology and variable clinical presentation. In some cases, SAS appears as hypertrophic cardiomyopathy with obstruction due to the accompanying systolic anterior motion of the mitral valve. A 46-year-old male with dizziness for several months presented in the outpatient department. Two-dimensional transthoracic echocardiography demonstrated a slightly hypertrophic left ventricle with normal systolic function without wall-motion abnormalities. Just below the aortic valve, a linear structure protruding from the septum side and the left-ventricular outflow tract (LVOT) side of the mitral valve was confirmed, which was causing a significant pressure gradient (mean and maximum of 91 mmHg and 138 mmHg, respectively). A diagnosis of SAS with subaortic membrane was made, and surgical myomectomy and subaortic membrane removal surgery were performed. Postoperative transthoracic echocardiography did not show flow acceleration through the LVOT, nor a significant pressure gradient across the aortic valve. This case report highlights the clinical significance of SAS with subaortic membrane, which can be confused with aortic stenosis of other etiology.

## 1. Introduction

Subaortic stenosis (SAS) is a rare heart disease in adults with an unclear etiology and variable clinical presentations. SAS usually develops during the first decade of life and might appear as an isolated lesion or in association with other congenital heart diseases [1]. SAS is most commonly described as a fibromuscular ring of tissue, but can also present as an incomplete shelf or ridge-like structure. The location of this structure can range from just below the aortic valve where it sometimes fuses with the leaflets, to lower within the LVOT where it can become attached to the anterior mitral valve leaflet. SAS usually follows a progressive course characterized by significant left-ventricular outflow tract (LVOT) obstruction, left-ventricular hypertrophy, and aortic valve destruction with subsequent regurgitation. In some cases, SAS appears as hypertrophic cardiomyopathy (HCM) with obstruction due to the accompanying systolic anterior motion of the mitral valve [2]. Although surgical treatment is successful in most cases, the recurrence rate is high, so continuous follow-up is required. We present a case of subaortic stenosis with subaortic membrane that was treated with surgical myomectomy and subaortic membrane removal surgery.

## 2. Case Presentation

A 46-year-old male presented to the outpatient department with dizziness he had been experiencing for several months. His medical history was unremarkable except for irritable bowel syndrome for years, and his vital signs were stable. Physical examination revealed a systolic murmur at the right-upper sternal border. Electrocardiography showed normal sinus rhythm with left-ventricular hypertrophy. Coronary computed tomography angiography showed no significant coronary artery stenosis, but a subaortic fibrotic membrane was observed.

**Figure 1:**
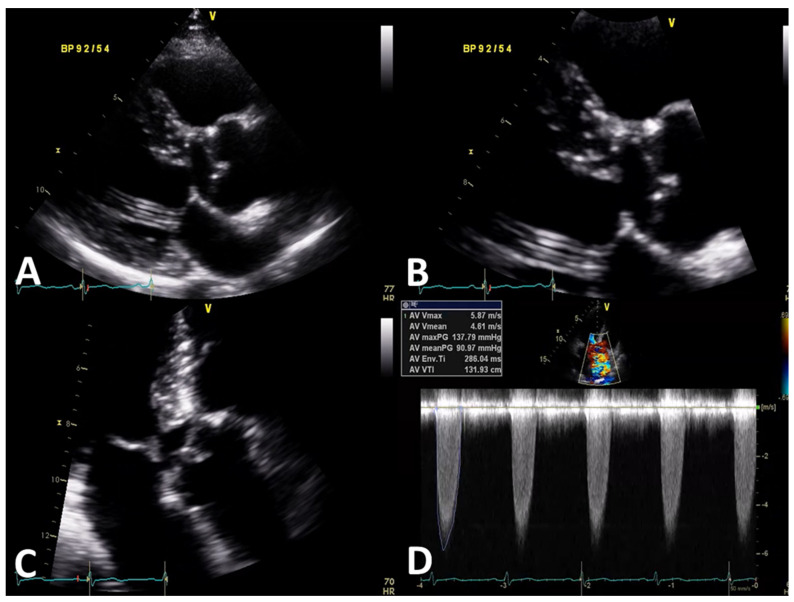
Transthoracic echocardiographic image showing a parasternal long-axis view (**A**,**B**), apical four-chamber view of the aortic valve (**C**), and continuous-wave Doppler across the aortic valve (**D**).

Two-dimensional transthoracic echocardiography demonstrated a normal-sized, slightly hypertrophic left ventricle with normal systolic function without wall-motion abnormalities. Just below the aortic valve, a linear structure protruding from the septum side and the LVOT side of the mitral valve was identified. Significant flow acceleration caused by these structures in the LVOT was seen, with a mean and maximal pressure gradient of 91 mmHg and 138 mmHg, respectively (Figure 1, Supplementary Videos S1 and S2). The aortic valve was tricuspid with a thickened leaflet, and mild aortic regurgitation was observed. Upon further inspection, dilatation of the ascending aorta with a diameter of 47 mm was detected. The patient was diagnosed as SAS with subaortic membrane, and surgical treatment was planned.

**Figure 2:**
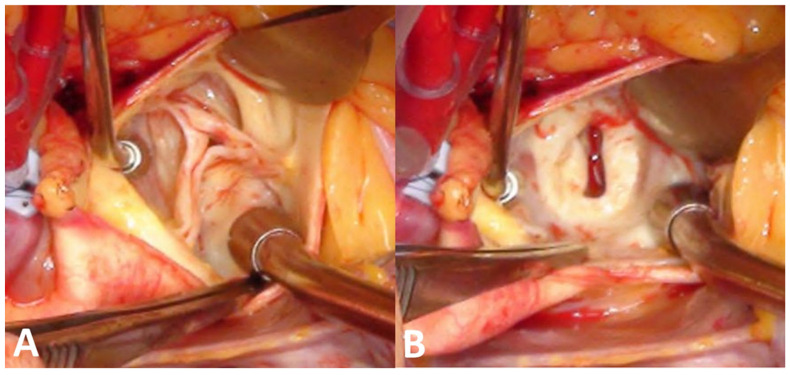
Surgical images of the aortic valve (**A**) and subaortic membrane (**B**).

**Figure 3:**
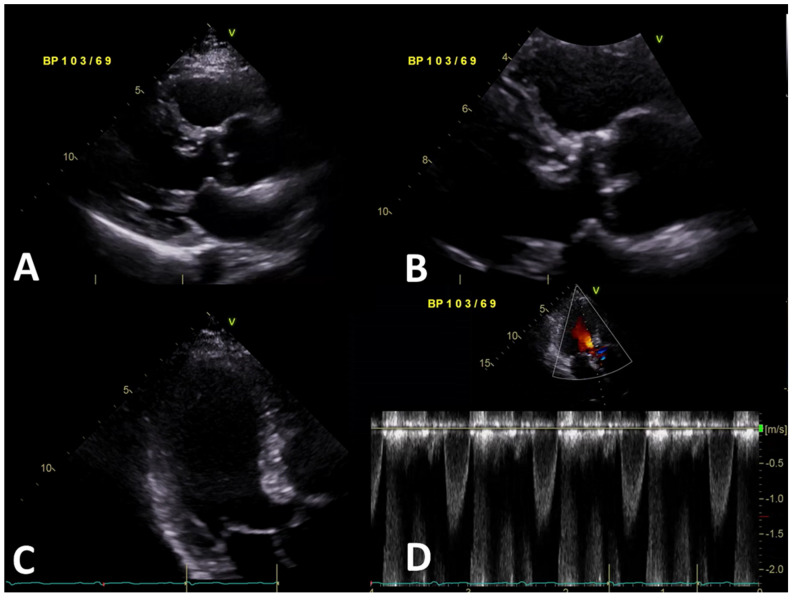
Transthoracic echocardiographic image showing a parasternal long-axis view (**A**,**B**), apical four-chamber view of the aortic valve (**C**), and continuous-wave Doppler across the aortic valve (**D**) after subaortic membrane removal surgery.

With the patient under general anesthesia, the aorta was opened transversely at the sino-tubular junction. Below the tricuspid aortic valve, an opening resembling a narrow gap made by the subaortic membrane was confirmed (Figure 2). The subvalvular membranes were resected, and a septal myomectomy was performed to enlarge the LVOT. Because there was an ascending aortic enlargement, ascending aorta wrapping was also performed. The pathology report of the resected membrane revealed myxoid degeneration with fibrosis. Postoperative transthoracic echocardiography did not show flow acceleration of the LVOT nor a significant pressure gradient across the aortic valve (Figure 3, Supplementary Videos S3 and S4). The patient was discharged in stable condition.

## 3. Discussion

In this case, we reported on a case of a patient with SAS caused by subaortic membrane who was successfully treated by surgical removal. Subaortic stenosis is a form of LVOT obstruction that usually presents in the first 10 years of life as a progressive disease and is rare in adults with an unclear etiology [3]. In SAS, risk factors such as morphological LVOT abnormalities (e.g., sharp aortoseptal angle, subphysiologic aortic annulus diameter, and large aortic valve-mitral valve separation distance) are known to increase the risk of endocarditis [4]. Subaortic stenosis may be associated with other congenital anomalies, especially membranous ventricular septal defect that can occur in ≤65% of cases [1]. Other congenital anomalies include bicuspid aortic valve, coarctation of the aorta, and patent ductus arteriosus [5,6].

Patients with subaortic stenosis can range from asymptomatic to varying degrees of symptoms, including dyspnea at rest or with exertion, palpitation, chest pain, or syncope. SAS generally tends to progress slowly, with an annual increase in the gradient across the stenotic lesion. Without appropriate surgical intervention, SAS can progress to left ventricular hypertrophy and dysfunction, aortic regurgitation, endocarditis, arrythmias, and death. Echocardiography is preferred to diagnose SAS and to characterize the anatomy of the subaortic lesion, assess LVOT involvement, determine the dimensions and function of the LV, and determine the integrity of the aortic and mitral valves. Differentiating SAS from other causes of LVOT obstruction, especially HCM, can be difficult because some patients with SAS develop asymmetrical septal hypertrophy and secondary dynamic subaortic obstruction. The pressure gradient of subaortic stenosis may be underestimated or overestimated in the presence of a ventricular septal defect [3].

Currently, there are no established medical therapies, such as balloon dilation, to reverse or stop the progression of SAS. Thus, for patients with significant obstruction, surgical intervention is appropriate, but surgery can be considered at a lower pressure gradient if LV systolic dysfunction occurs due to left ventricular remodeling. Also, left ventricular remodeling is known to be associated with poor prognosis after surgery [6]. Surgical therapy for SAS consists of correction of the obstruction, which may involve simple membrane removal, extensive ring resection with or without myectomy, or a Konno procedure [7,8,9]. The timing of the surgery varies, with recommendations ranging from early operation to longer periods of observation depending on the patient’s characteristics. Survival after SAS surgery has been shown to be excellent. However, the LVOT gradient still increases slowly over time. Reoperation for recurrent discrete subaortic stenosis is commonly needed, with a reoperation rate between 6% and 30% [10]. The risk of reoperation may be due to inadequate resection at the first operation, yet recurrent obstruction may appear despite the adequacy of surgical excision. The risk of recurrent SAS is related to younger age at diagnosis and surgery, smaller aortic annulus, proximity of the obstruction to the aortic valve, and a higher preoperative peak LVOT gradient [11,12,13]. Females are have a 1.5 times higher risk of recurrence compared to males. This increased risk might be associated with a smaller LVOT anatomy in females [14].

In conclusion, subaortic stenosis remains a rare and clinically challenging diagnosis in adults, and its clinical presentation can closely mimic HCM with obstructive physiology. Therefore, a combination of imaging modalities is often needed to diagnose SAS with subaortic membrane and accompanying heart diseases. Although the standard treatment for subaortic stenosis is surgical correction, the recurrence rate is high, so regular echocardiographic follow-up is required.

## Data Availability

Data are contained within the article.

## Supplementary Materials

The following supporting information can be downloaded at: https://www.mdpi.com/article/10.3390/jcdd9020036/s1. Video S1: Transthoracic echocardiographic image showing a parasternal long-axis view of the aortic valve; Video S2: Transthoracic echocardiographic image showing an apical four-chamber view of the aortic valve; Video S3: Transthoracic echocardiographic video showing a parasternal long-axis view of the aortic valve after subaortic membrane removal surgery; Video S4: Transthoracic echocardiographic video showing an apical four-chamber view of the aortic valve after subaortic membrane removal surgery.
